# Tunica vaginalis or dartos as second layer coverage for distal and mid-shaft penile hypospadias, quo vadis?

**DOI:** 10.1007/s00345-024-05419-x

**Published:** 2025-01-18

**Authors:** Mohamed Ramez, Abdelwahab Hashem, Mahmoud Bazeed, Mohamed S. Dawaba, Tamer E. Helmy

**Affiliations:** 1 Urology Department, Urology and Nephrology Center, Mansoura University, Mansoura, Egypt; 2 Urology Department, 30, June Urology and Nephrology Centre, Ismailia, Egypt; 3 Urology Department, Met Ghamr Urology and Nephrology Hospital, Dakahlia, Egypt

**Keywords:** Dartos flap, Tunica vaginalis flap, Hypospadias, Fistula, Healing

## Abstract

**Purpose:**

To compare between the dartos and tunica vaginalis flaps as covering layers in denovo distal or mid-shaft penile hypospadias underwent tubularized incised plate (TIP) repair.

**Methods:**

This is a single-center, randomized trial was for denovo distal or mid-shaft penile hypospadias. Children with history of orchiectomy, orchiopexy and inguinal hernia repair were excluded. Eighty-eight patients were divided into two groups: the first used dartos flap (DF), while the second used tunica vaginalis flap (TVF). The primary outcome was to assess the incidence of urethrocutaneous fistula. The secondary outcome was to assess cosmetic outcome using paediatric penile perception score (PPPS) and hypospadias objective scoring evaluation (HOSE).

**Results:**

Baseline demographic and clinical characteristics showed no statistically significant. Median operative time (IQR) was 100 (90, 120) and 145 (140, 150) minutes in in DF and TVF Groups, respectively (p < 0.001). Urethrocutaneous Fistula was detected in 9 (20.9%) in DF group and 2 (4.9%) in TVF group (p 0.029). Meatal stenosis occurred in 3 (7%) in DF group and 2 (4.9%) in TVF group. Penile torque was diagnosed in one (2.4%) in TVF group. There was no significant difference in total PPPS score (p = 0.076), however, there was a significant difference in total HOSE score in the favour to TVF group (p = 0.024). At 12 months, testicular ascent occurred in 0% and 3 (7.3%) in DF and TVF groups, respectively (p = 0.071).

**Conclusion:**

Compared to dartos flap, tunica vaginalis flap significantly helps in reduction of fistula rate. However, it has significant more operative time.

## Introduction

Hypospadias is one of the most common congenital male birth anomalies which is characterized by abnormal ventral placement of the urethral meatus with reported prevalence rate of > 50/10,000 total birth [1]. Hypospadias repair is considered a challenging procedure that needs well-trained skilled experienced surgeons to correct it. Treatment includes different surgical techniques such as tubularization, advancement techniques and the use of grafts and flaps. The goal of hypospadias repair is to achieve a penis that is both functionally and cosmetically acceptable [2].

Urethrocutaneous fistula (UCF) is the most frequent complication after hypospadias repair. It continues to be a frustrating complication for both surgeons and families as it does not only occur but also recurs, sometimes requiring many procedures in the same patient with reported incidence range from 0 to 50% [3]. Second layer coverage of the neourethra has been shown to significantly decrease rate of fistula. Various procedures have been developed to avoid occurrence of fistulae [4].

Dartos flap (DF) has been utilized as a covering layer. This includes mobilizing a vascularized pedicle flap from the dorsal aspect of the penis and rotating it ventrally. This may cause penile torsion, so some buttonholed and brought it ventrally to lie over the neourethra. However, the dorsal prepuce tends to suffer blood supply deterioration when the dorsal dartos is aggressively dissected from the skin to provide an abundant DF and to avoid penile rotation [5, 6].

Tunica vaginalis flap (TVF) is available, has good vascularity and is not affected by penile disorders. It provides an abundant large flap to wrap the urethroplasty or fistula closure with a very high success rate. However, it may be difficult to obtain a long pedicle because the proximal extent of the tunica vaginalis is sometimes limited anatomically [7].

Different studies have been conducted on both dartos flap and tunica vaginalis flap as covering layers in hypospadias repair, two of them comparing the two flap techniques in recurrent cases [8, 9] and only three comparing them in primary repair [10–12] including only one prospective comparative but non-randomized study [11].

Because the quality of studies comparing dartos and tunica vaginalis flaps were mostly retrospective comparative studies, a prospective randomized trial is needed to evaluate the two flap techniques.

## Patients and methods

This prospective randomized controlled, registered clinical trial (ClinicalTrials.gov identifier NCT05123833) was conducted between October 2021 and August 2022 at specialized pediatrics urology unit of tertiary urology and nephrology center. Children diagnosed with hypospadias who were scheduled for tubularized incised plate (TIP) repair were assessed. Legible subjects fulfilling inclusion criteria were included in a clinical trial and their parents were asked to sign an informed consent form.

Eligible patients were children aged six months or more, diagnosed with distal or mid-shaft penile hypospadias with good urethral plate amenable for TIP repair. Recurrent cases and patients with history of orchiectomy, orchiopexy and inguinal hernia repair were excluded.

Patients were allocated into two groups. In dartos flap group, 44 children underwent hypospadias repair using DF as a covering layer for neourethra. While in tunica vaginalis flap group, 44 children underwent hypospadias repair using TVF for the same purpose. The randomization process was carried out using closed envelope technique in a 1:1 ratio. Patients were assigned to the study groups on the day of surgery.

All surgeries were performed, as day-surgery procedures, under general anesthesia and a caudal epidural block. Perioperative antibiotics (intravenous cephalexin) were administered with the induction of anesthesia. A glanular stay suture is placed in the midline along the long axis of the penis. A circumferential subcoronal incision is done. The penis is degloved strictly between the dartos fascia layer and Buck’s fascia. The neurovascular bundle is used as the landmark of the dissection plane. Dissection starts from the dorsum of the penis and not from the ventral aspect, as otherwise the actual plane of dissection may be missed; maintaining the plane minimizes bleeding. A tourniquet is used. After degloving, the degree of chordee is assessed by artificial erection with mild traction in the long axis through the stay suture in the glans. A small incision is made in the midline from within the meatus to the end of urethral plate. Urethral tubularization is done with running subcuticular polyglycolic sutures (6/0) over 6 Fr urethral catheter.

For the dartos flap group, after completing urethral tubularization, coverage of the neourethra is done using a dorsal or ventral dartos flap, according to the surgeon perefernce. A dorsal well-vascularized dartos flap was harvested from excessive dorsal preputial and penile hypospadiac skin, and transposed ventrally by a buttonhole manoeuvre. A ventral based well-vascularized dartos flap was harvested from both the right and the left side of the urethral corporal groove, and the flap was turned up.

For tunica vaginalis flap group; testis and spermatic cord are delivered into the operative field by blunt and sharp dissection of penoscrotal junction after degloving of the penis. Tunica vaginalis and spermatic fascia are dissected from the right testis. A strip of tunica vaginalis (15–20 mm wide) is separated from the parietal layer of the tunica vaginalis of right testis by sharp dissection. Near head of epididymis, only tunica vaginalis is incised in the transverse axis, preserving spermatic fascia to create an island of tunica vaginalis based on the pedicle of spermatic fascia by a similar dissection for separating the hernial sac from spermatic cord in orchidopexy for an undescended testis using absorbable suture.

**Fig. 1:**
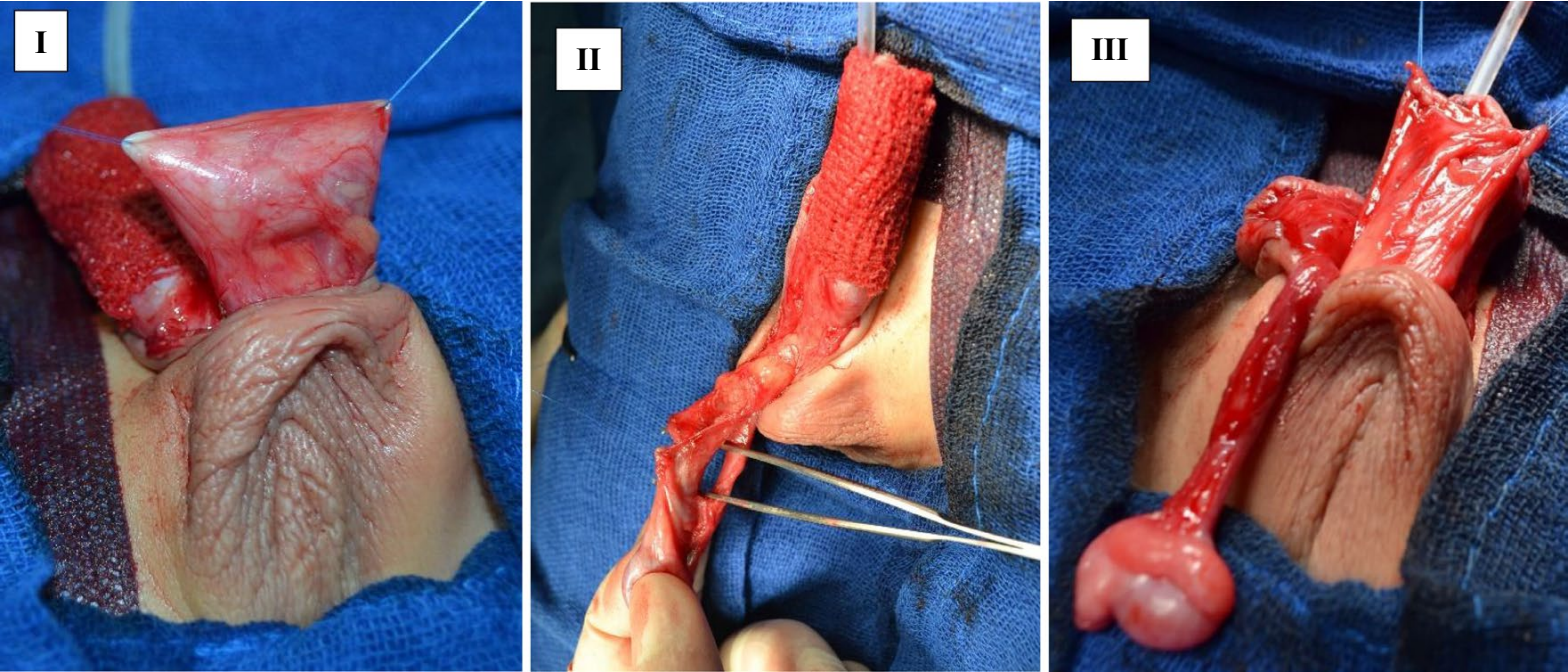
Tunica vaginalis flap (TVF) steps; **I**. Delivering the testis into operative field, **II**. Dissection of TV and spermatic cord from testis, **III**. Wrapping of TV over the neourethra

Spermatic fascia is dissected from the spermatic cord towards the superficial inguinal ring to keep the pedicle tension‐free. After completing urethral tubularization, TVF is wrapped over the neourethra (Fig. 1) by fixing the tunica vaginalis, spermatic fascia with Buck's fascia and the bases of glanular wings around the neourethra using 5/0 polyglycolic sutures. The testis is placed in the scrotum with orchiopexy. The glans is then reconstructed over the tunica vaginalis pedicled wrap with no tension in the suture line using 5/0 polyglycolic sutures. All surgeries were performed by experienced two surgeons who spent at least three years of pediatric urology fellowship. This fellowship must be preceded by completion of five years in general urology residency.

Post-operatively, A self-adherent elastic wrap (3 M Coban™) was lightly applied to the penis for 3 days and urethral catheter for 10 days. Parents were instructed to apply topical antibiotic ointment with diaper changes after dressing removal. Postoperative analgesic, antispasmodic and oral antibiotics were prescribed for one week. Patients were evaluated at outpatient clinic 1 month and 3 months postoperatively. Fistula prevalence was recorded and parents’ reported cosmetic outcome was evaluated using the pediatric penile perception score (PPPS) and the hypospadias objective scoring evaluation (HOSE). The investigator together with the parents completed the following two questionnaires.

The primary outcome was to assess the incidence of UCF within the first three months after surgery. The secondary outcome was to document patients’ parents cosmetic outcome using PPPS and HOSE. A per-protocol analysis is performed and includes all randomized patients who completed their follow up. The null hypothesis assumed there is no difference between DF and TVF in decreasing the incidence of UCF post hypospadias repair.

Calculation of the sample size: using G*power program and chi-square test to calculate sample size, assuming type I statistical error of 5% and type II statistical error of 20%, to obtain a power of 80% and based upon difference of about 30% between both techniques in Sharma study [9], the total calculated number were 80 patients, plus 8 patients for dropout. Data were statistically analyzed using the Statistical Package for Social Sciences (SPSS Inc., Chicago, IL, USA, version 21). Independent sample t-test, and χ2 (chi-squared) test, were used for comparison between the two groups, as appropriate. “P-value ≤ 0.05” was used to refer to results as statistically significant.

## Results

**Fig. 2:**
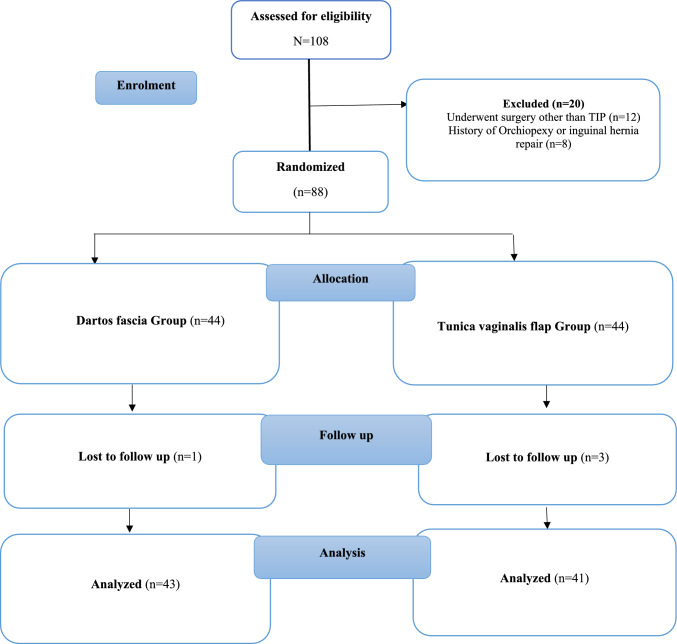
CONSORT flowchart

Out of 108 hypospadias patients, 88 patients met the inclusion criteria and were eligible to take part in the study. 44 patients underwent TIP repair using dartos flap and the other 44 patients underwent TIP repair using tunica vaginalis flap. Out of the patients who were randomized, four patient lost follow up and 84 patients were included in the final analysis. (Fig. 2).

Patients’ age median, interquartile range (IQR) at time of surgery was 4 (3, 5) and 4 (3, 5) in dartos flap and tunica vaginalis flap Groups, respectively. Body mass index (BMI) median (IQR) at time of surgery was 17.2 (15.6, 18.3) and 18.5 (16, 19.7) in Dartos flap and Tunica vaginalis flap Groups, respectively. In dartos flap group, 21 patients (48.8%) had coronal hypospadias, 12 patients (27.9%) had distal penile hypospadias and 10 patients (23.3%) had mid penile hypospadias. In tunica vaginalis flap groups, 13 patients (31.7%) had coronal hypospadias, 16 patients (39%) had distal penile hypospadias and 12 patients (29.3%) had mid penile hypospadias. Chordee was diagnosed in 6 patients (14%) in dartos flap group, and 4 patients (9.8%) in tunica vaginalis flap group. There was no statistically significant difference between the two groups regarding their baseline demographic and clinical characteristics. (Table 1).

**Table 1:** Baseline patients’ demographic and clinical characteristics

	Dartos flap group	Tunica vaginalis flap group	P value
Number of patients	43	41	
Age, years, median (IQR)	4 (3, 5)	4 (3, 5)	0.556
Body mass index, kg/m^2^, median (IQR)	17.2 (15.6, 18.3)	18.5 (16, 19.7)	0.062
Hypospadias site N (%)			
Coronal	21 (48.8%)	13 (31.7%)	0.27
Distal penile	12 (27.9%)	16 (39%)	
Mid penile	10 (23.3)	12 (29.3%)	
Associated chordee N (%)	6 (14%)	4 (9.8%)	0.553

Chordee was corrected after degloving of the penis, apart from that; 2 cases in dartos flap group and 1 case in tunica vaginalis flap group, needed dorsal plication of the tunica albuginea. Median operative time (IQR) was 100 (90, 120) and 145 (140, 150) minutes in in dartos flap and tunica vaginalis flap groups, respectively (p < 0.001) (Table 2).

**Table 2:** Perioperative parameters and postoperative follow up

	Dartos flap group	Tunica vaginalis flap group	P value
3-months follow up			
Operative time, minutes, Median (IQR)	100 (90, 120)	145 (140, 150)	< 0.001
Intraoperative chordee plication N (%)	2 (4.7)	1 (2.4)	0.59
Complications			
Meatal stenosis	3 (7)	2(4.9)	
Stricture	1 (2.3)	0	
Bleeding	1 (2.3)	0	0.20
Tourque	0	1(2.4)	
Edema	0	3 (7.3)	
Pain	0	2 (4.9)	
Urethrocutaneous fistula N (%)	9 (20.9%)	2 (4.9%)	0.029
Total PPPS score, median (IQR)	14 (11, 17)	15 (13, 16)	0.076
Total HOSE score, median (IQR)	15 (13, 16)	15 (15, 16)	0.024
12-months follow up			
Urethrocutaneous fistula N (%)	9 (20.9%)	2 (4.9%)	0.029
Testicular ascent	0	3 (7.3%)	0.071

One case (2.3%) in dartos flap group presented with significant bleeding and was admitted and managed conservatively. Three cases (7.3%) in Tunica vaginalis flap group complained of sever scrotal swelling. Those cases were managed by reassurance and oral alpha-amylase twice daily. Catheter slippage occurred in one case (2.4%) in tunica vaginalis flap group on the 5th day postoperative. Reinsertion of the catheter was done. We had only 3 patients had skin infections, two children in dartos flap group and one child in tunica vaginalis flap group (p = 0.59), with no reported skin flap necrosis in this trial.

**Fig. 3:**
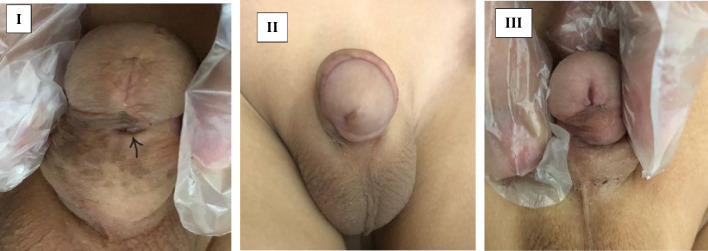
Some of our reported complication; **I**. Urethrocutanous fistula (arrow), **II**. Penile torsion, **III**. Meatal stenosis

At 3-months, urethrocutaneous fistula was detected in 9 cases (20.9%) in dartos flap group and 2 cases (4.9%) in tunica vaginalis flap group (p 0.029). Meatal stenosis occurred in 3 cases (7%) in dartos flap group and 2 cases (4.9%) in tunica vaginalis flap group. Meatotomy was performed in 4 cases, while one case was managed by regular dilatation. Penile torque was diagnosed in one case (2.4%) in tunica vaginalis flap group which had torque degree less than 30⁰ and no further intervention was indicated (Fig. 3).

There was no significant difference observed between the two groups in terms of the total PPPS score (p = 0.076). However, there was a significant difference between the two groups in terms of total HOSE score in the favor to tunica vaginalis flap group (p = 0.024).

At 12 months, testicular ascent occurred in 0% and 3 (7.3%) in DF and TVF groups, respectively (p = 0.071). Urethrocutaneous fistulas still the same as ocuured in 3 months as no new cases was discovered. Children with testicular ascent occurred at the level of scrotum neck and managed conservatively.

## Discussion

Despite progress in surgical techniques for repairing hypospadias, complications still arise. One of the most frequent complications is urethrocutaneous fistula [13]. The occurrence of urethrocutaneous fistula (UCF) can be reduced by tissue interpositioning between the neourethra and skin. Various methods for providing vascularized soft tissue cover to the neourethra have been described [12].

An advancement that contributed to the improvement of TIP repair results was the additional coverage of the neourethra using a vascularized inner preputial dartos flap. However, this dissection requires skill and there are chances that the outer preputial skin becomes hypovascular after dissection of DF If the preputial skin gets devascularised while raising the dartos flap, eventually it can result in ventral skin necrosis and can lead to increased incidence of UCF [11].

On the other hand, the tunica vaginalis flap is known for its robust blood supply, as it has its own independent blood supply and does not negatively impact the blood flow to the outer preputial foreskin [10]. The unpopularity of TVF might be explained by the more difficult technical approach [9]. This flap is tunneled through the scrotal tissue to reach the neourethra which may however increase morbidity. The greatest risk is injury of the testicular vasculature or vasa. In rare cases in which hypospadias is associated with other surgically correctable pathologies requiring testicular exposure, such as hydrocele, inguinal hernia, an undescended testis, there is a logical indication to expose the testis to correct this and simultaneously harvest a tunical flap for urethral coverage during hypospadias repair [4].

Several authors reported better outcomes when using TVF when compared to DF. The use of the tunica vaginalis as an interposition layer was first reported by Snow et al. in 1995, with a 9% fistula rate [7]. However, Snodgrass reported zero fistula rate in 14 patients suffering from proximal hypospadias who were treated using double-layered urethroplasty and tunica vaginalis cover [14].

There are excellent results for TVF, with superiority over DF for primary and repeat cases and for fistula repair. The use of TVF in repairing recurrent proximal UCF has shown to be more effective compared to local flap cover, with an overall success rate of 80% compared to a success rate of only 50% if a local flap is utilized [9].

Additionally, studies conducted by Guralnick et al. and Landau et al. have shown that the use of tunica vaginalis in hypospadias repair results in low rates of fistula formation and other complications [15, 16]. Hamid et al. reported fistula rate of 2.85% with TVF cover [17]. Routh et al. described the use of TVF combined with the use of operative microscope and reported that the UCF rate was 0% with only a 2.2% complication rate such as scrotal hematoma and abscess formation [18].

In a systematic review by Fahmy et al., Among 244 patients aged 9 months to 22 years with follow-up of 1 month to 17 years after TVF, only five (2.0%) developed fistula, compared to 170 of 4034 patients (4.2%) after DF [4]. To reduce the incidence of UCF complications, a method of covering the neourethra with a double layer of dartos flap had been developed. In a recent meta-analysis, the use of a double dartos flap layer lessen fistula formation in cases of distal penile hypospadias compared to single layer of single layer [19].

There are a limited number of studies that have compared the two methods of soft tissue cover. A study by Chatterjee et al. in a a multi-institutional study had a fistula rate 0% for cases with TVF coverage, while it was 15–20% for cases with DF coverage [20]. In a comparative non-randomized study by Dhua et al., the rate of fistula formation was 12% with the use of a DF and 0% with the use of a TVF [11].

To the best of our knowledge, our study is the first randomized study comparing dartos flap and tunica vaginalis flap. Our study reveals that TIP repair with TVF has statistically significant better outcomes in terms of fistula rate which was 4.9% compared to 20.9% when DF was used; however, we think, theoretically speaking, if one case added case could change the significance. Also, TVF required longer operative time.

TVF procedure typically requires additional surgical incision, which might result in additional complications such as hematoma, infection, beside dehiscence. In this trial, three children (7.3%) in Tunica vaginalis flap group complained of sever scrotal swelling, managed conservatively.

The advantages of TVF are multiple. A very low UCF rate, uniformly good blood supply, easy operative access to tunica vaginalis, especially in the hands of an experienced hypospadias surgeon who is well-versed with the surgical anatomy of the scrotum, testis and cord structures makes this technique very useful and versatile for repair of hypospadias. One theoretical concern has been the risk of damage to vas and testicular vessels. The possibility of injuring the testicular artery and vas deferens is low because the dissection is done in a more distal direction over the tunica vaginalis that surrounds the testis [17].

Meatal stenosis occurred in 3 cases (7%) in dartos flap group and 2 cases (4.9%) in tunica vaginalis flap group with insignificant p-value (p = 0.2). Snodgrass reckoned that failure to incise the plate deeply enough or sewed the plate too far distally, could inducing meatal stenosis which could be the cause in our trial. Elbakry supposed that the double flap layer was a risk factor for meatal stenosis due to urethral compression [19].

Another possible complication is post-operative torque which is probably due to short length of the pedicle resulting in subsequent downward traction of the glans towards the side where TVF had been harvested. This complication can be avoided by ensuring that the flap is of adequate length and no cremasteric fibers are included in the flap [21].

Testicular ascent is another feared complication of using TVF however; this can be avoided by performing orchiopexy. Remaining processus vaginalis adherent to the spermatic cord or developed adhesion of the spermatic cord in the inguinal canal due to wide dissection during might cause the postoperative testicular ascent [22]. In our trial, orchidopexy is performed using absorbable suture. Besides, the distal position of the neo-meatus with long tract of the flap plays a role in this ascent. This needs other trial investigating this point. At 12 months, testicular ascent occurred in 3 (7.3%) in TVF group, respectively (p = 0.071). This did not reach the statistical significance; however, we think, theoretically speaking, if one case added case could change the significance.

In our study, parents were asked to complete the two questionnaires together with the investigator. Total PPPS score was comparable between both groups. Total HOSE score was slightly higher in tunica vaginalis flap group. This may attributed to HOSE score has an item assess the number and fistula presence.

The limitation of our study was the short follow-up period and the fact that the surgeries were not performed by a single surgeon. The age of children at the time of surgery was older than other published trials. Also, the sample size was calculated on non-randomised controlled trials, and if one complication occurred plus or minus our data, the significance could be change in fistula or testicular ascent. So, other randomised controlled trials are warranted.

## Conclusion

Compared to dartos flap, tunica vaginalis flap significantly helped in reduction of fistula rate. However, it had significant more operative time.

## Data Availability

No datasets were generated or analysed during the current study.
